# Single-Institution Early Experience With a New, Smooth, Opaque, and Round Breast Implant Over a 2-Year Period

**DOI:** 10.1093/asjof/ojad090

**Published:** 2023-10-12

**Authors:** Zhi Yang Ng, Calum Honeyman, Taimur Shoaib

## Abstract

**Background:**

The ideal breast implant does not exist and the choice of implant for breast augmentation is largely based on what surgeons think will be best for their patients.

**Objectives:**

To evaluate the preliminary results of a new, smooth, round, and opaque breast implant (PERLE, GC Aesthetics; Dublin, Ireland) from a single-center UK aesthetic practice.

**Methods:**

Retrospective cohort study of all patients undergoing breast implant surgery with PERLE at the authors' center between January 2021 and December 2022. Outcomes data such as rates of capsular contracture, infection, revision surgery, and synchronous mastopexy were analyzed.

**Results:**

Of the 385 patients identified, 374 (97.1%) had PERLE implants placed by 3 surgeons for primary (*n* = 290) and secondary breast augmentation (*n* = 21), and augmentation-mastopexy (*n* = 63). Capsular contracture occurred in no cases, infection in 1 (0.2%), and revision surgery in 21 patients (5%). The incision used was always submammary, unless a synchronous mastopexy was performed; implants were placed in the subglandular/subfascial plane in the majority of cases (85.3%), and the rest were dual plane (14.7%). Eight revisions were performed in patients undergoing breast augmentation (due to implant displacement in 6 patients, and hematoma and infection in 1 patient each). Fourteen revisions were performed in those undergoing augmentation-mastopexy. The average follow-up time was 18 months.

**Conclusions:**

The authors' early, single-center experience with PERLE implants suggests a safety profile and overall complication rate that is comparable with other modern implants. They will continue to monitor the safety and effectiveness of PERLE and discuss the reasons and evolution in the choice of breast implant.

**Level of Evidence: 4:**

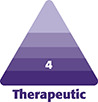

Modern breast implant surgery is considered to be very safe, with a recent study from Italy demonstrating no perioperative mortality in almost 100,000 such patients over a period of 8 years.^1^ Yet, the ideal breast implant still does not exist. Surgeons choose breast implants based on a shared decision-making process with patients, taking into account their individual ideals, requirements, and the best available evidence.^2^ An ideal implant should not be carcinogenic, remain within its fitted location, be inert, last a lifetime, represent good value for money, not affect surrounding tissues, allow radiological and clinical examination without interference, and should not cause complications in both the short and long term. Different breast implants are available on the market in the United Kingdom, and newer ranges of implants are being released by manufacturers.^3^

Breast implant texturing has been the topic of debate in recent years. Highly textured implants are associated with anaplastic large cell lymphoma (ALCL), although the overall risk remains low^4^ and smooth implants have, in some series, been associated with higher risks of capsular contracture.^5^ Newer implants with minimal texturing are now available and have been so for a few years. Some of these implants contain radiofrequency (RF) chips, whereas others do not. The International Standards Organization (ISO) classes implants into whether they are smooth or textured and at least one of the ISO smooth implants must have minimal texturing (PERLE Smooth Opaque Round implant, GC Aesthetics, Dublin, Ireland). The Motiva Ergonomix range of implants (Establishment Labs, Alajuela, Costa Rica) are also minimally textured but contain an RF chip (Figure 1). Overall, smooth implants do not seem to be associated with ALCL, and so these implants, at least in theory, should have minimal to zero risk of developing ALCL in patients.^6^

**Figure 1.**
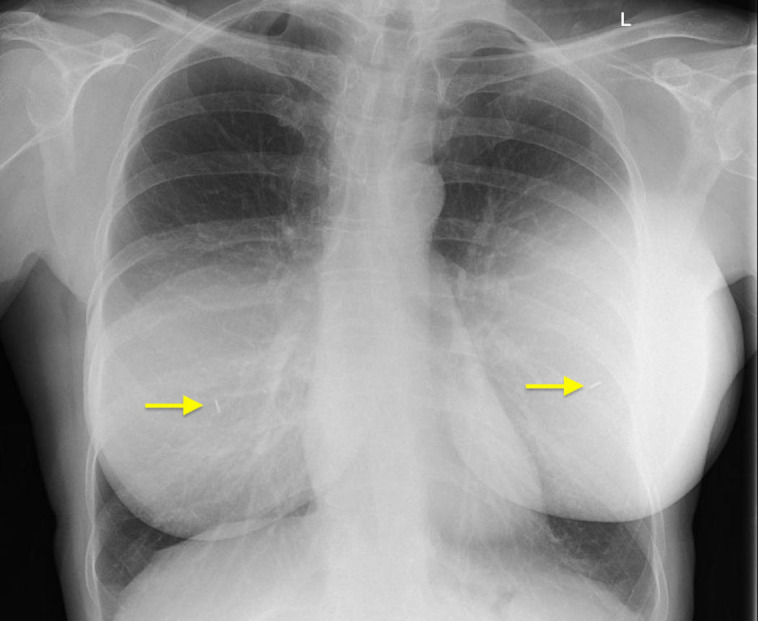
Radiofrequency chip visible on chest X-ray (highlighted by arrows).

The senior author (T.S.), and his colleagues at the same private practice hospital (the Glasgow Day Surgery Centre), started using the GC Aesthetics PERLE range of implants in early 2020. Here, only aesthetic surgery is performed and not reconstruction. These implants were chosen on a background of previous use of Nagor Impleo implants (GC Aesthetics) that contain the same gel as GC Aesthetics PERLE implants. The senior author (T.S.) subsequently used Allergan textured implants (Irvine, CA, USA) for several years, followed by Allergan smooth implants, Motiva Ergonomix implants, and now GC Aesthetics PERLE implants. Of note, Nagor was rebranded as GC Aesthetics recently. Allergan textured implants were removed from the UK market in 2019,^3^ and Allergan smooth implants were, in the senior author's practice, associated with higher and unacceptable rates of capsular contracture (unpublished data from internal audit, not dissimilar to recent reports^7^). Patients with Motiva Ergonomix implants also complained about interference with radiological imaging by the RF chips after undergoing aesthetic breast surgery with these implants.^8^

Because breast implants can be associated with both short- and long-term unusual findings or discoveries such as ALCL,^4^ and more recently, squamous cell carcinoma,^9^ the authors performed an analysis of 2 years' worth of patient data who had GC Aesthetics PERLE implants placed to evaluate the early experience of this implant in patients undergoing aesthetic breast surgery.

## METHODS

### Study Design

A retrospective chart review of all patients undergoing aesthetic breast surgery at our center between January 2021 and December 2022 was undertaken. Only patients who had the PERLE implant placed were included. Corresponding data on the operating surgeon, the pocket in which the implant was placed, whether the patient underwent any concomitant mastopexy procedure, follow-up period, and whether the patient underwent further surgery following implant insertion, were collected and analyzed. The number of surgeries, the surgeon involved, and the type of, and reason for, revision surgery were also noted. All patients were otherwise followed up till July 2023. Written informed consent was also obtained preoperatively from all patients in the current study, which was designed in accordance with the principles of the Declaration of Helsinki.

### The PERLE Breast Implant—Smooth, Round and Opaque

According to the current ISO 14607:2018 classification system, the round PERLE breast implant is considered to have a smooth outer surface with a surface roughness of 5 μm. However, it differs from typical smooth breast implants by virtue of its biomimetic topography, which is believed to improve the biocompatibility of its surface to mitigate against bacterial growth, inflammation, and hence, the risk of capsular contracture.^10^ Additionally, the PERLE breast implant is “opaque” in having a multilayered elastomer shell to minimize leakage and diffusion of the encapsulated 6th generation silicone gel.^3^ This is also a means to differentiate from traditional smooth implants (ie, less microns) and a consequence of the reverse manufacturing technique used to create the texture of the surface, which is salt free. Three profile ranges are available—moderate (MR), high (HR), and extra-high (EHR)—with a base width and projection ranging from 8.1 to 13.4 cm and 3.4 to 6.6 cm, respectively. As with standard practice, the choice of implant size and projection was based on the patients' desires and preferences, as well as their suitability for particular implants based on physical examination by the operating surgeon.

### Study Demographics

All patients who were above 18 years of age and had undergone aesthetic breast surgery with placement of the PERLE implant at a private practice hospital by the senior author (T.S.) and his colleagues during the study period above were included. The aesthetic breast surgery operations performed included primary and secondary breast augmentation, and augmentation-mastopexy in both unilateral (*n* = 3, 0.9%) and bilateral procedures.

### Surgical Approach

All procedures were carried out under general anesthesia as day case procedures, which is the standard practice at our center. The incisions used were always submammary, unless a synchronous mastopexy was performed, for which the incision would then be designed as a mastopexy/reduction pattern. The planes of implant placement were subglandular/subfascial or dual-plane pockets. The rest of the procedure was otherwise performed in standard fashion with the use of perioperative antibiotics, nipple guards, antibiotic/antiseptic lavage of the pocket, and dipping of the implant into antiseptic solution (Betadine) before delivery into the breast pocket through a funnel for final placement. Funnels and nipple shields were not used in augmentation-mastopexy cases, and not all surgeons in the group used a funnel (Video).

### Postoperative Follow-Up

Postoperatively, patients were discharged home in a support bra once deemed safe. Follow-up visits were then arranged for at 1, 6 weeks, and 6 months thereafter. The policy of our center is to perform revisional surgery without charge on patients who would like to do so if the surgeon feels that there is a possibility of improvement.

### Statistical Analysis

Descriptive statistics were employed in the present study using standard computer statistical software. Numbers were presented as means with range as appropriate and percentages accordingly.

## RESULTS

**Figure 2.**
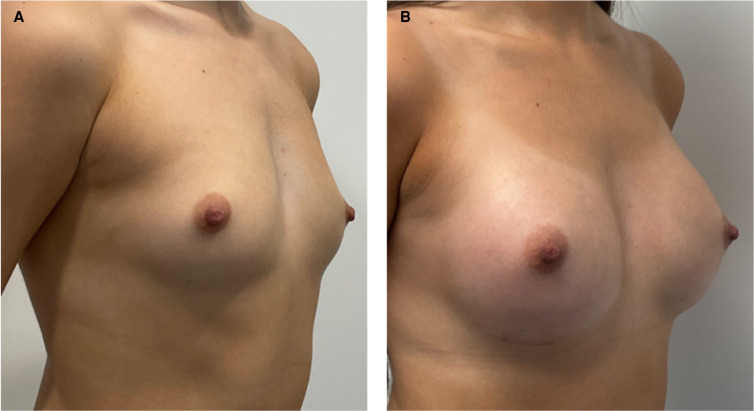
Representative results in a 32-year-old female patient following subglandular breast augmentation with 280 cc moderate (MR) profile PERLE implants (GC Aesthetics) through a submammary incision: (A) preoperative and (B) postoperative (at 6 months) views.

**Figure 3.**
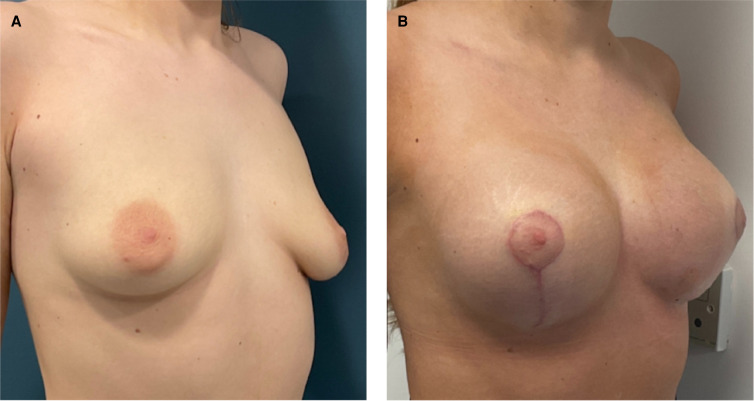
Representative results in a 36-year-old female patient following augmentation-mastopexy with 340 cc moderate (MR) profile PERLE implants (GC Aesthetics) with a mastopexy/reduction approach: (A) preoperative and (B) postoperative (at 6 months) views.

Between January 2021 and December 2022, 385 patients underwent aesthetic breast surgery at our center and 374 (97.1%) had the PERLE breast implant placed on them. All patients (mean age = 32 years, range 19 to 62) identified themselves as female, with the exception of one, who self-identified as gender fluid (Table 1). The majority of patients underwent bilateral procedures (99.1%) through a subglandular/subfascial approach (85.3%), with primary breast augmentation (77.6%) (Figure 2) being the most commonly performed, followed by augmentation-mastopexy (16.8%) (Figure 3). Overall, a total of 745 PERLE breast implants were placed, with the majority being either MR (45.5%) or HR (36.8%) profile (Table 2).

**Table 1.** Study Demographics

Average age (years)	32.0 (range, 19-62)
Sex	
Female	373 (99.7%)
Gender fluid	1 (0.3%)
Procedure	
Primary breast augmentation	290 (77.6%)
Augmentation mastopexy	63 (16.8%)
Secondary breast augmentation	21 (5.6%)
Average follow-up (months)	18 (range, 6-30)

**Table 2.** Details of Aesthetic Breast Surgery Procedures

Details	Values
Laterality	
Unilateral	3 (0.8%)
Bilateral	371 (99.2%)
Different between sides	19 (5.2% of 374 total)
PERLE implant (GC Aesthetics) profile	
Total number implanted	745
MR (range, 220-430 cc)	339 (45.5%)
HR (range, 175-550 cc)	274 (36.8%)
EHR (range, 210-575 cc)	132 (17.7%)
Incision	
Inframammary fold	620 (83.2%)
Mastopexy/reduction	125 (16.8%)
Plane	
Subglandular/subfascial	635 (85.3%)
Dual plane	110 (14.7%)

EHR, extra-high; HR, high; MR, moderate.

### Type and Rate of Complications

**Figure 4.**
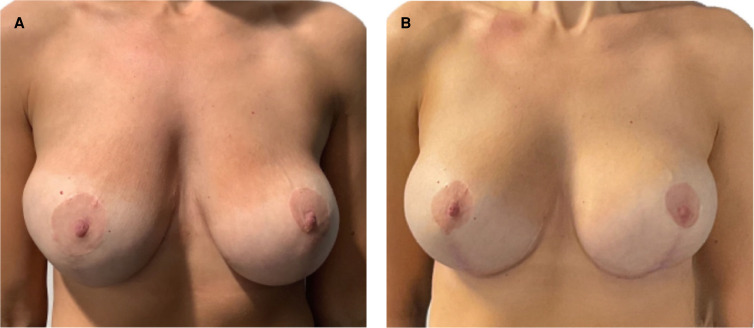
Illustrative example of a 42-year-old female patient who underwent augmentation-mastopexy with subsequent drifting of the implant inferiorly and laterally from tissue stretch requiring revision mastopexy and pocket adjustment: (A) preoperative (at 6 weeks after the index procedure) and (B) postoperative (at 6 weeks after the revision procedure) views.

Postoperative follow-up averaged 18 months (range, 6-30) in this cohort of patients. The overall complication rate was 5.6% (21/374 patients), with scar revision and/or lower pole stretch (*n* = 14) being the most common, followed by implant displacement (*n* = 6); all required revisional surgery (Table 3). Of note, 67% (14/21) of the complications were in patients who underwent augmentation-mastopexy (Figure 4). To date, no cases of capsular contracture, seroma, or implant rupture have been reported in this cohort of patients. Only one patient expressed dissatisfaction with her final result, and requested for a larger size.

**Table 3.** Details of Postoperative Complications in Patients Requiring Revision

Complications	Value
Scar revision	9
Lower pole stretch	3
Implant displacement	6
Hematoma	1
Infection	1
Dissatisfaction with final size	1
Total	21 (5.6% of 374 patients)

## DISCUSSION

The PERLE breast implant is a new implant that has been introduced to the market. The main advantage of this implant is that it is considered smooth by ISO standards, and therefore, the theoretical risk of ALCL should be eliminated. The implant texturing is made by inverting the silicone shell from the mandrel when the implant is being manufactured. The gel used within the implant is one that has been present within the Nagor Impleo range of breast implants for many years. Capsular contracture rates in smooth implants have, in some studies, been shown to be higher than textured implants and is one of the factors behind resistance to their use in the United Kingdom.^11,12^ It seems reasonable then for a surgeon to choose an implant that is smooth, based on empirical evidence of next to no ALCL rates.^13,14^ Therefore, when such a device became available (ie, GC Aesthetics PERLE), a discussion of its use with the patient was warranted.

Capsular contracture is arguably the most common long-term complication after breast implant insertion, but its true incidence is unknown.^15^ Most cases of capsular contracture will manifest themselves within a short time frame, such as 1 to 2 years, and therefore, it is not unreasonable to assess the various choices of implants for this time frame, at least initially. In 1 study, it was reported that 16% of patients developed capsular contracture within a follow-up period of 1.6 years after implantation.^16^ Adams et al reported a lower capsular contracture rate of 1.8% in primary augmentation cases with a mean follow-up of 14 months.^17^ In a recent single-surgeon study with a similar set up as this study (ie, private practice setting in the United Kingdom), 0% and 2.3% capsular contracture rates were reported at an average of 20.2 months follow-up for primary and secondary breast augmentation, respectively, with a microtextured silicone gel–filled implant; at last follow-up, it was reported that 91.2% of breasts were rated as Baker I.^18^ However, it should be noted that the validity of the Baker classification has been called to question, with the authors of a recent study suggesting poor interobserver reliability and observer agreement, with up to 12% discordance in more than one category of clinical assessment of capsular contracture.^19^

Although the follow-up period in the present study is similar, with a mean of 18 months (range, 6 to 30), capsular contracture can still develop many years after implantation and for many reasons that remain poorly understood.^20^ For example, one patient in this study required a revision from a different implant manufacturer to PERLE breast implants following a road traffic accident in which the seatbelt caused trauma to the chest wall, swelling, and subsequent capsular contracture on the injured side. This same patient then developed a hematoma after surgery. Although it is reassuring to note that no incidences of capsular contracture have been reported in the present study, a longer term follow-up will undoubtedly yield cases of capsular contracture. To illustrate, microtextured (ie, smooth) round implants had a 0% capsular contracture rate with a mean of 20.2 months follow-up in a study by Tanner,^18^ compared with Hong et al, who used similar implants with an incidence rate of 2.1% at 4 years' follow-up.^21^ This may be attributed to the reduced foreign body response and fibrosis when the implant surface roughness was approximately 4 μm, as demonstrated in animal models,^22,23^ and in turn, decreased capsular contracture rates in both retrospective cosmetic^24^ and reconstructive^25^ cohorts. Otherwise, we have endeavored to utilize modern and established strategies such as antibiotic irrigation^26^ to minimize bacterial contamination, which seems to protect against the risk of capsular contracture, postoperative surgical site infection, and ALCL,^27^ as described in the Surgical Approach section.

Unsurprisingly, complication rates and the need for revisional surgery were higher in patients undergoing augmentation-mastopexy compared with implant-based surgery alone, due to an already compromised ptotic breast.^28^ The revision rate in the present study is comparable to other published case series.^29,30^ Of note, revisional surgery was required in 22% (14/63) of patients undergoing augmentation-mastopexy, mainly due to tissue stretch following the process of postoperative tissue expansion. As a result, implants may consequently sit lower or more lateral compared with the ideal position, which contrasts with the demands of patients undergoing cosmetic surgery, who are looking for upper and medial pole fullness in many cases (Figure 4). Breast support material was not used routinely in our practice, however. Interestingly, a systematic review reported that recurrent ptosis was the most common reason for revisional surgery following one-stage augmentation-mastopexy,^31^ whereas in another recent case series, this was due to implant size exchange.^32^

Complications in the present study were not likely to be directly associated nor attributable to the PERLE breast implant itself. This is not surprising, considering the relatively short follow-up period in this study, but it is also reassuring at the same time to confirm that indeed, the new implant's characteristics are not a barrier to continued use both at our center and elsewhere. To reiterate, we have chosen to use the PERLE breast implant because of its likely negligible risk of ALCL, the presumed low risk of capsular contracture, as demonstrated in our results thus far (although limited by subjective assessment), and the track record of safety with regard to surface characteristics. Our results, however, are limited by the retrospective nature of the present study and the relatively short-term follow-up. Additionally, our results were limited to a single center and 3 surgeons, which indicates the need for carrying out future studies with larger, multicenter cohorts and longer follow-up times for obtaining more confirmatory evidence of superiority, or otherwise, of our chosen implant. Indeed, the above is supported by a long-term prospective study with 25 years’ follow-up in 1529 women by Handel et al, who reported a relatively high rate of reoperation not long after the primary surgery, mainly due to capsular contracture. Most tellingly, they demonstrated that the cumulative risk of developing capsular contracture increased progressively over time.^33^

## CONCLUSIONS

In this study, we shared the evolution of our experience by using various types of breast implants in our aesthetic practice before settling on the PERLE round, smooth, and opaque breast implant because of its meeting and satisfying various criteria of both our surgeons and patients; therefore, we are happy to continue using this implant. Nevertheless, we are obliged to continue active surveillance in our patients, and hence, the present study, which reports a short-to-medium-term follow-up following the placement of the new-to-market PERLE breast implants.

## Supplementary Material

ojad090_Supplementary_Data
